# A nomogram for the prediction of short-term mortality in patients with aneurysmal subarachnoid hemorrhage requiring mechanical ventilation: a post-hoc analysis

**DOI:** 10.3389/fneur.2023.1280047

**Published:** 2024-01-08

**Authors:** Qing Mei, Hui Shen, Jian Liu

**Affiliations:** ^1^Department of Neurology, Beijing Pinggu Hospital, Beijing, China; ^2^Department of Interventional Neuroradiology, Sanbo Brain Hospital, Capital Medical University, Beijing, China; ^3^Department of Functional Neurosurgery, Zhujiang Hospital, Southern Medical University, The National Key Clinical Specialty, The Engineering Technology Research Centre of Education Ministry of China, Guangdong Provincial Key Laboratory on Brain Function Repair and Regeneration, Guangzhou, China

**Keywords:** aneurysm subarachnoid hemorrhage, mechanical ventilation, prediction, mortality, nomogram

## Abstract

**Background:**

Aneurysmal subarachnoid hemorrhage (aSAH) is a devastating stroke subtype with high morbidity and mortality. Although several studies have developed a prediction model in aSAH to predict individual outcomes, few have addressed short-term mortality in patients requiring mechanical ventilation. The study aimed to construct a user-friendly nomogram to provide a simple, precise, and personalized prediction of 30-day mortality in patients with aSAH requiring mechanical ventilation.

**Methods:**

We conducted a post-hoc analysis based on a retrospective study in a French university hospital intensive care unit (ICU). All patients with aSAH requiring mechanical ventilation from January 2010 to December 2015 were included. Demographic and clinical variables were collected to develop a nomogram for predicting 30-day mortality. The least absolute shrinkage and selection operator (LASSO) regression method was performed to identify predictors, and multivariate logistic regression was used to establish a nomogram. The discriminative ability, calibration, and clinical practicability of the nomogram to predict short-term mortality were tested using the area under the curve (AUC), calibration plot, and decision curve analysis (DCA).

**Results:**

Admission GCS, SAPS II, rebleeding, early brain injury (EBI), and external ventricular drain (EVD) were significantly associated with 30-day mortality in patients with aSAH requiring mechanical ventilation. Model A incorporated four clinical factors available in the early stages of the aSAH: GCS, SAPS II, rebleeding, and EBI. Then, the prediction model B with the five predictors was developed and presented in a nomogram. The predictive nomogram yielded an AUC of 0.795 [95% CI, 0.731–0.858], and in the internal validation with bootstrapping, the AUC was 0.780. The predictive model was well-calibrated, and decision curve analysis further confirmed the clinical usefulness of the nomogram.

**Conclusion:**

We have developed two models and constructed a nomogram that included five clinical characteristics to predict 30-day mortality in patients with aSAH requiring mechanical ventilation, which may aid clinical decision-making.

## Introduction

Stroke has become the leading cause of death and disability, with an escalating burden in China (1). Aneurysmal subarachnoid hemorrhage (aSAH) is a devastating type of hemorrhagic stroke, representing 3.1% of all strokes (2), with a 30-day mortality rate of up to 36% (3). Despite improved neurosurgical techniques and management and a decline in the global incidence of aSAH, 30-day mortality has remained unchanged over the last decades (4). Approximately one-third of the survivors recovered fully and returned to independent living after bleeding (5).

The establishment of relevant prediction models in terms of short-term mortality may support clinicians in their treatment recommendations. In addition, prediction models may provide a convenient and graphic tool for patients and relatives. Currently, several models have been developed to estimate the mortality in patients with aSAH. However, few predictive models have evaluated the short-term mortality in patients with aSAH requiring mechanical ventilation.

In the present study, we focused on patients with aSAH requiring mechanical ventilation to identify predictors and build a nomogram to predict the risk of 30-day mortality.

## Materials and methods

This is a post-hoc analysis of a previously completed retrospective cohort study conducted in a university hospital intensive care unit in Montpellier, France. In the present study, a total of 236 confirmed patients with aSAH requiring mechanical ventilation were enrolled between January 2010 and December 2015 (6). Patients with iatrogenic aneurysm ruptures and those without follow-up were excluded. Based on critical condition on admission (Glasgow coma scale under 9), patients with aSAH were treated with sedation and mechanical ventilation to prevent secondary brain damage and maintain body temperature between 36 °C and 38 °C. All treatments followed the international guidelines (7). No additional patient informed consent was required because the data used in this study were publicly available from a public database (8) and patient information was anonymous.

The variables of patients with aSAH requiring mechanical ventilation collected for further analysis were as follows: age, sex, hypertension, tobacco use, alcohol abuse, diabetes, cardiovascular disease, aneurysm location, Glasgow coma scale (GCS) on admission, simplified acute physiology score II (SAPS II), World Federation of Neurosurgical Societies (WFNS) score, presence of intracerebral hemorrhage (ICH), Fisher score, early brain injury (EBI), external ventricular drain (EVD), rebleeding, angiographic vasospasm, length of mechanical ventilation (LMV), delayed cerebral ischemia (DCI), and 30-day mortality. The specific variables are defined as follows: EBI: Patients with cerebral hypodensities on CT scans who experienced neurological deterioration, raised ICP within 48–72 h, or were brain dead before day 5 were defined as having EBI. Rebleeding: An increase in subarachnoid blood between two CT scans. Angiographic vasospasm: at least one-third reduction in arterial diameter on CTA or digital subtraction angiography (9). DCI: delayed clinical deterioration due to ischemia or a delayed cerebral infarction discovered by CT or MRI scans (10). 30-day mortality: measured at 30 days after admission.

### Statistical analysis

Before data analysis, predictor variables in the retrospective cohort were inspected for missing values. To obtain five imputation datasets, we used the “mice” package of R (seed = 0879) to address missing values with multiple imputations.

Continuous variables were presented as the mean ± SD or median (IQR), and categorical variables were expressed as numbers (percentages). Continuous variables were performed using an unpaired, two-tailed t-test, or Mann–Whitney U-test as appropriate. Categorical variables were compared using the χ^2^ test or Fisher’s exact test. To facilitate clinical application, we dichotomized continuous variables (SAPS II, GCS, and WFNS) based on the cutoff values derived from the receiver operating characteristic (ROC) curve analysis (11).

The least absolute shrinkage and selection operator (LASSO) regression technique (R package glmnet) was used to select the most useful predictive features from high-dimensional data (12). Prediction model A was constructed by integrating independent clinical risk factors. The clinical intervention factor was added to model A to develop prediction model B based on the features selected from the LASSO regression. Moreover, two forest plots (R package forest model) were constructed to present significant predictors and odds ratios for correlations with the risk of 30-day mortality.

A nomogram (R package regplot) of 30-day mortality in aSAH patients requiring mechanical ventilation was formulated based on model B. The discriminatory capacity of the model was determined by calculating the area under the curve (AUC) (13). The bootstrapping method (resampling = 1,000) was employed for internal validation (14). The calibration of the model was evaluated by using a calibration plot and the Hosmer–Lemeshow test. The Brier score was chosen for overall performance, as it can capture both discrimination and calibration (15). Decision curve analysis (DCA) was then performed to determine the clinical practicability of the nomogram based on the standardized net benefits at different threshold probabilities in the retrospective cohort (16, 17).

A two-tailed value of *p* of <0.05 was considered statistically significant in our study. R software (version 4.1.0; R Foundation for Statistical Computing, Vienna, Austria, https://www.r-project.org) was used for statistical analysis. All analyses were reported according to the Transparent Reporting of a Multivariable Prediction Model for Individual Prognosis or Diagnosis (TRIPOD) guidelines (14).

## Results

### Participant characteristics

The demographic and clinical characteristics of study participants after imputation are summarized in Table 1. In total, 68 patients (28.81%) died within 30 days after admission. Of the enrolled patients, the median age was 56 years, 62% were female, and 46% had intracerebral hemorrhage. In most cases, the neurological severity of the condition on admission was critical, with 90% of the patients having a WFNS grade of IV–V, 78% having a Fisher’s scale of IV, and a median GCS of 7.0 (IQR,4.0–9.0). When the patients were admitted to the ICU within 24 h, the median score on the SAPS II severity scale was 43 (IQR,35–50).

**Table 1:** Participant characteristics.

Characteristic	Overall, *N* = 236^a^	Survivors, *N* = 168	Non-survivors, *N* = 68	*p*-value^b^
Age(year), Median (IQR)	56 (46, 64)	55 (46, 64)	57 (46, 63)	0.8
Sex, n (%)				0.8
Female	147 (62%)	104 (62%)	43 (63%)	
Male	89 (38%)	64 (38%)	25 (37%)	
Hypertension, *n* (%)	82 (35%)	60 (36%)	22 (32%)	0.6
Tobacco use, *n* (%)	76 (32%)	59 (35%)	17 (25%)	0.13
Alcohol abuse, *n* (%)	20 (8.5%)	15 (8.9%)	5 (7.4%)	0.7
Diabetes, *n* (%)	8 (3.4%)	7 (4.2%)	1 (1.5%)	0.4
Cardiovascular disease, *n* (%)	40 (17%)	27 (16%)	13 (19%)	0.6
Aneurysm location, *n* (%)	197 (83%)	142 (85%)	55 (81%)	0.5
GCS, *n* (%)	7.0 (4.0,9.0)	7.0 (4.0, 9.0)	5.0 (3.0, 8.0)	0.009
SAPS II, Median (IQR)	43 (35, 50)	40 (34, 48)	47 (39, 52)	0.002
WFNS score, *n* (%)				0.016
3	24 (10%)	18 (11%)	6 (8.8%)	
4	97 (41%)	78 (46%)	19 (28%)	
5	115 (49%)	72 (43%)	43 (63%)	
Fisher score, *n* (%)				0.8
1	1 (0.4%)	1 (0.6%)	0 (0%)	
2	5 (2.1%)	4 (2.4%)	1 (1.5%)	
3	46 (19%)	35 (21%)	11 (16%)	
4	184 (78%)	128 (76%)	56 (82%)	
ICH, *n* (%)	109 (46%)	80 (48%)	29 (43%)	0.5
EBI, *n* (%)	169 (72%)	109 (65%)	60 (88%)	<0.001
EVD, *n* (%)	159 (67%)	122 (73%)	37 (54%)	0.007
Rebleeding, *n* (%)	34 (14%)	14 (8.3%)	20 (29%)	<0.001
Angiographic vasospasm, *n* (%)	84 (36%)	67 (40%)	17 (25%)	0.031
DCI, *n* (%)	64 (27%)	47 (28%)	17 (25%)	0.6
LMV(day), Median (IQR)	12 (6, 27)	20 (9, 36)	5 (2, 10)	<0.001

GCS, Glasgow coma scale; SAPS II, simplified acute physiology score II; WFNS, World Federation of Neurological Surgeons; ICH, intracerebral hemorrhage; EBI, early brain injury; EVD, external ventricular drain; DCI, delayed cerebral ischemia.

a Median (IQR); n (%) LMV: length of mechanical ventilation.

b Wilcoxon rank sum test; Pearson’s chi-squared test; Fisher’s exact test.

The neurological events observed in the ICU, in decreasing order of frequency, were EBI (72%), angiographic vasospasm (36%), delayed cerebral ischemia (27%), and rebleeding (14%). Moreover, 156 patients (67%) were diagnosed with hydrocephalus requiring EVD.

### A prognostic nomogram for 30-day mortality

The results of the univariate analysis are also summarized in Table 1. The univariate analysis identified the following significant variables for 30-day mortality after admission in aSAH patients between the survival and non-survival groups: GCS on admission (7.0 [IQR, 4.0–9.0] vs. 5.0 [IQR, 3.0–8.0]; *p* = 0.009), SAPS II (40 [IQR, 34–48] vs. 47 [IQR,39–52]; *p* = 0.002), WFNS score (*p* = 0.016), EBI (*p* < 0.001), EVD (*p* = 0.007), and rebleeding (p < 0.001).

**Figure 1:**
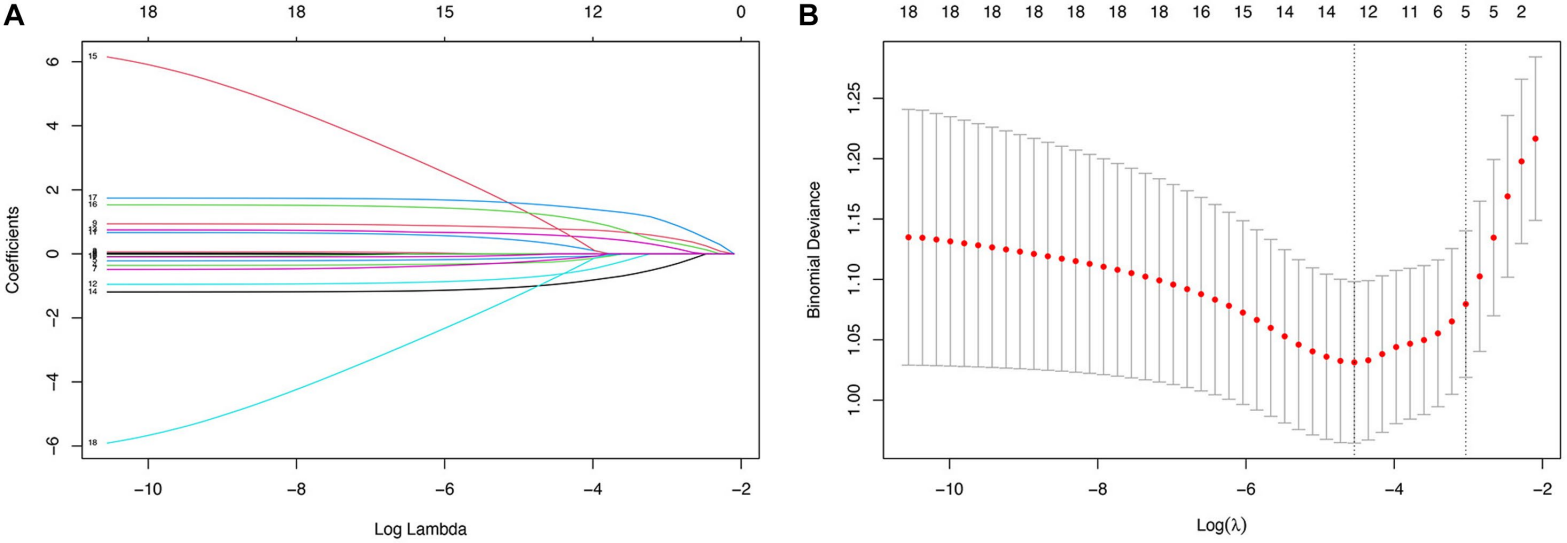
Predictor selection using the LASSO regression analysis with 10-fold cross-validation. **(A)** A coefficient profile plot was created against the log (lambda) sequence. **(B)** Tuning parameter (lambda) selection of deviance in the LASSO regression based on the minimum criteria and the 1-SE criteria.

According to the cutoff values derived from ROC curve analysis, GCS was dichotomized into equal to 4 and greater than 4, and SAPS II was dichotomized into less than 45 and 45–79. In all 18 variables collected from patients, five potential predictors were selected based on the LASSO regression model (Figure 1). These potential predictors included admission, GCS, SAPS II, rebleeding, EBI, and EVD. To develop an early prediction model for aSAH patients requiring mechanical ventilation, model A incorporated four clinical factors: GCS, SAPS II, rebleeding, and EBI. Then we added the clinical intervention factor EVD to model A to develop a new prediction model (model B) that is appropriate for clinical practice.

**Figure 2:**
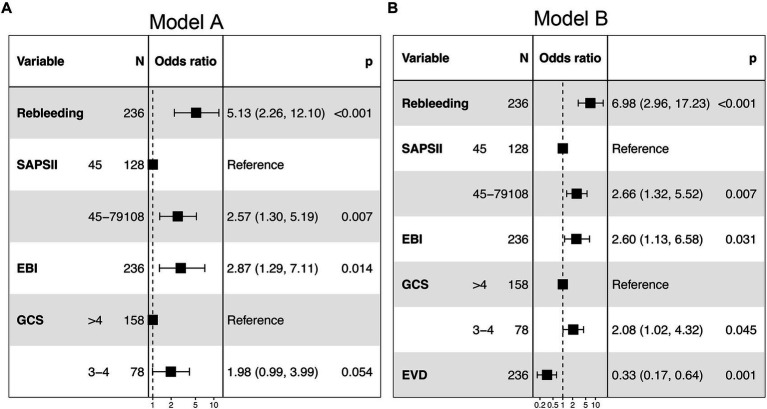
Forest plot of the two multivariable logistic regression models (model **A** and model **B**) for predicting the risk of 30-day mortality in patients with aSAH requiring mechanical ventilation. SAPS II, simplified acute physiology score II; EBI, early brain injury; EVD, external ventricular drain; GCS, Glasgow Coma Score.

**Figure 3:**
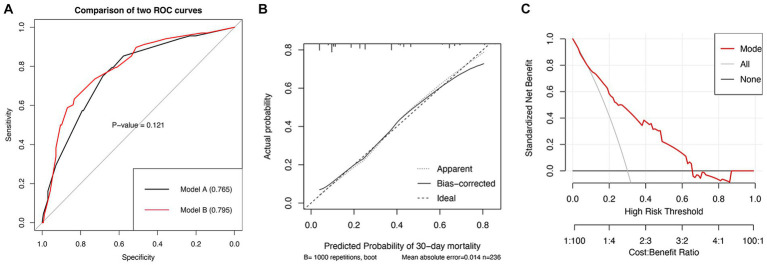
ROC curves **(A)** for comparisons of the AUC between model A and model B (value of *p* = 0.121).Calibration plot for the nomogram model **(B)**. The horizontal axis represents the nomogram-predicted probability of 30-day mortality, and the vertical axis represents the actual observed 30-day mortality. Decision curve **(C)** implicates the standardized net benefit with respect to the use of the nomogram. AUC, area under the curve.

**Figure 4:**
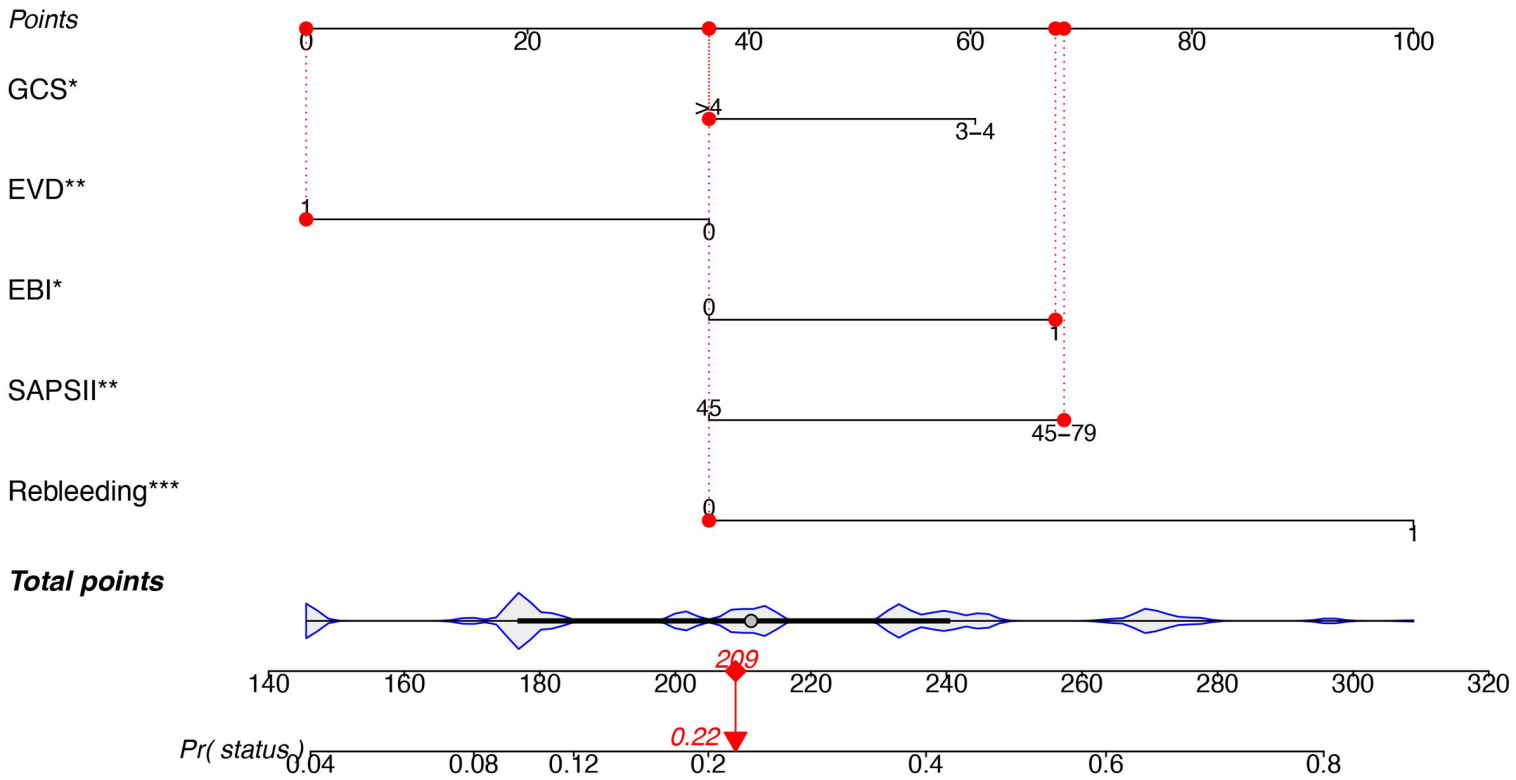
Nomogram predicting the risk of 30-mortality in patients with aSAH requiring mechanical ventilation. The nomogram is used by summing all points identified on the scale for each variable. The total points projected on the bottom scale indicate the probability of 30-day mortality. SAPS II, simplified acute physiology score II; EBI, early brain injury; EVD, external ventricular drain; GCS, Glasgow Coma Score.

The predictors related to the 30-day mortality of aSAH identified by multivariable logistic regression in the two models are demonstrated in the forest plots (Figure 2). The ROC (Figure 3A) was conducted to compare the discrimination performance of the models (*p* = 0.121). Moreover, by introducing the above five independent predictors, the 30-day mortality of aSAH risk nomogram was developed based on model B and is presented in Figure 4.

### Performance evaluation of the prognostic nomogram

In estimating 30-day mortality of aSAH, the satisfactory predictive performance of the nomogram was moderate to good accuracy (AUC: 0.795 [95% CI, 0.731–0.858]), for internal validation with bootstrapping (AUC: 0.780).

The calibration graph (Figure 3B) also revealed a good concordance between the nomogram-predicted probabilities and actual probabilities throughout the scale’s score range (Brier score = 0.158). The Hosmer–Lemeshow test yielded a non-significant *p*-value of 0.516.

To assess its clinical usefulness, the decision curve for the nomogram is demonstrated in Figure 3C. The decision curve indicated that in the current study, the nomogram was more accurate in predicting the risk of 30-day mortality when the risk threshold probability was between 10 and 64%.

## Discussion

In the present study, we conducted two prediction models to recognize the risk factors related to the 30-day mortality in patients with aSAH. Model A included variables that were easily available at admission or within 24 h, including GCS and SAPS II, and other parameters associated with poor prognosis, rebleeding, and EBI. Model B (model A + EVD) was constructed using five significant predictors identified by LASSO regression. These five variables were used to establish a prognostic nomogram based on model B that demonstrated good discrimination, goodness-of-fit, and clinical usefulness in identifying patients with a high risk of 30-day mortality. Moreover, more effort is needed to improve the model and make it more accurate and practical.

Several predictive models have been developed to estimate the prognosis of SAH patients (18–20). However, the predictive models for short-term mortality in patients with SAH requiring mechanical ventilation are rarely reported. Li et al. retrospectively analyzed 310 patients and found that age, lymphocyte, neutrophil, CRP, AST, and treatment were independent predictors of 3-month all-cause death (21). Then, they built a predictive model including the above predictors with an accuracy of 0.811. However, this model did not consider the severity of the disease on admission, which significantly affected the clinical outcomes. Another predictive model recently reported by Zhou et al. has enrolled 150 patients with WFNS IV–V and established a nomogram with an accuracy of 0.909 (22). However, all patients analyzed in their study were treated by clipping, which may bias the results.

Due to the advancement of diagnostics and treatment strategies, case fatality rates have decreased after adjustment for age in recent decades (23). In this study, the 30-day mortality (28.81%) fell within the ranges reported in other studies (3, 4, 24). Still, it was higher than the rates reported in population-based studies (24), owing to poor-grade SAH (25) and clinical conditions on admission.

In our study, lower admission GCS and SAPS II were predictors of 30-day mortality, which had been confirmed in previous studies. Maragkos et al. (26) observed that an admission GCS score of 3–8 was one of the risk factors for predicting mortality and functional outcomes in patients with aSAH. Consistent with another retrospective cohort study, the requirement for mechanical ventilation, a higher APACHE III score, and lower GCS were found to be associated with lower survival to discharge (27). Moreover, some studies showed that physiologic parameters, such as hypoxemia, metabolic acidosis, electrolyte abnormalities (28) and cardiovascular instability (29) were independent predictors of mortality in SAH patients. SAPS II scores had all these factors in their automated calculation tables.

In our study, rebleeding was a particularly strong independent predictor of 30-day mortality in aSAH, which was consistent with other studies. Zhao et al. (30) conducted a multicenter prospective registry on the long-term outcomes of patients with poor-grade aSAH and found that aneurysm rebleeding was independently associated with mortality (OR 25.03, *p* < 0.001) at 12 months. In a nationwide population-based study conducted in 2018, rebleeding was treated as a binary variable within a multivariate regression model. The analysis revealed a significant association between rebleeding and in-hospital mortality (31). The cumulative incidence of rebleeding was reported to range between 4 and 17% of aSAH patients during the first 3 days (32) and mainly occurred within the first 6 h after ictus (33). Therefore, to reduce the risk of rebleeding and mortality, early or ultra-early aneurysm repair was recommended for patients with poor-grade SAH (25, 33, 34). Antifibrinolytic therapy was considered a protective approach and was safe to use within 72 h after aSAH (35). However, a Cochrane review did not support the use of antifibrinolytic therapy, although results on short-term treatment were promising (36). Interdisciplinary decision-making approaches may be reasonable for patients with poor-grade SAH (25).

EBI is considered to be the primary factor associated with high mortality after SAH (37), which refers to the immediate brain damage that occurs within 72 h after bleeding (38). Li et al. developed a new prognostic model based on the EBI in patients with aneurysmal subarachnoid hemorrhage with similar accuracy to our study (39). In recent clinical trials, the incidence of vasospasm was reduced without improving functional outcomes (40). Therefore, the focus of experimental and clinical research shifted toward the EBI evoked by SAH. Management strategies aimed to mitigate EBI by providing an adequate blood supply to the brain and normalizing pathophysiological parameters, although no specific treatment was currently available (41). It has been reported that the pathogenesis of EBI is strongly associated with intracranial hypertension and decreased cerebral blood flow. Our study confirmed that EVD is an effective approach to reducing intracranial hypertension after aneurysm repair and may, in turn, reduce 30-day mortality in poor-grade SAH.

The discriminative power of the nomogram was moderate to good accuracy with AUC (0.795 [95% CI, 0.731–0.858]), which was an acceptable power. Furthermore, the novel nomogram provided valuable information to help clinicians make decisions based on five common and easily accessible clinical parameters. Patients with aSAH requiring mechanical ventilation might benefit from the individualized treatment.

This predictive model has several limitations. First, this nomogram was built based on a single centre. Second, a prospective study was required to further confirm the nomogram’s reliability. Third, most patients presented with a severely poor clinical condition requiring mechanical ventilation, which limited the extent of the results to other populations. Finally, our study lacked detailed data on pulmonary diseases, neurogenic pulmonary edema, and pulmonary infections, which could potentially influence short-term mortality. Despite these limitations, this study was the first to develop a nomogram for predicting 30-day mortality in patients with aSAH requiring mechanical ventilation.

## Conclusion

We have developed a nomogram for predicting 30-day mortality in patients with aSAH requiring mechanical ventilation, which may aid clinical decision-making. The model included admission GCS, SAPS II, rebleeding, EBI, and EVD. Although our model has demonstrated good discrimination ability and reasonable performance, it still requires external validation.

## Data availability statement

The original contributions presented in the study are included in the article/supplementary material, further inquiries can be directed to the corresponding authors.

## Ethics statement

Ethical approval was not required for the study involving humans in accordance with the local legislation and institutional requirements. Written informed consent to participate in this study was not required from the participants or the participants' legal guardians/next of kin in accordance with the national legislation and the institutional requirements.

## Author contributions

QM: Conceptualization, Formal analysis, Methodology, Writing – original draft. HS: Formal analysis, Methodology, Writing – original draft. JL: Conceptualization, Methodology, Writing – original draft, Writing – review & editing.

## References

[1] Tu W-J Zhao Z Yin P Cao L Zeng J Chen H. Estimated burden of stroke in China in 2020. JAMA Netw Open (2023) 6:e231455. doi: 10.1001/jamanetworkopen.2023.1455, PMID: 36862407 PMC9982699

[2] Tu W-J Wang L-D. China stroke surveillance report 2021. Mil Med Res (2023) 10:33. doi: 10.1186/s40779-023-00463-x, PMID: 37468952 PMC10355019

[3] Sandvei MS Mathiesen EB Vatten LJ Müller TB Lindekleiv H Ingebrigtsen T. Incidence and mortality of aneurysmal subarachnoid hemorrhage in two Norwegian cohorts, 1984-2007. Neurology (2011) 77:1833–9. doi: 10.1212/WNL.0b013e3182377de3, PMID: 22049205

[4] Øie LR Solheim O Majewska P Nordseth T Müller TB Carlsen SM. Incidence and case fatality of aneurysmal subarachnoid hemorrhage admitted to hospital between 2008 and 2014 in Norway. Acta Neurochir (2020) 162:2251–9. doi: 10.1007/s00701-020-04463-x, PMID: 32601806 PMC7415018

[5] Hop JW Rinkel GJ Algra A van Gijn J. Case-fatality rates and functional outcome after subarachnoid hemorrhage: a systematic review. Stroke (1997) 28:660–4. doi: 10.1161/01.str.28.3.660 9056628

[6] Sauerbrei W Royston P Binder H Van Calster B Wynants L Verbeek JFM. Long-term outcome in patients with aneurysmal subarachnoid hemorrhage requiring mechanical ventilation. PLoS One (2021) 16:e0247942–13. doi: 10.1371/journal.pone.0247942, PMID: 33711023 PMC7954305

[7] Connolly ESJ Rabinstein AA Carhuapoma JR Derdeyn CP Dion J Higashida RT. Guidelines for the management of aneurysmal subarachnoid hemorrhage: a guideline for healthcare professionals from the American Heart Association/american Stroke Association. Stroke (2012) 43:1711–37. doi: 10.1161/STR.0b013e3182587839 22556195

[8] Chalard K Szabo V Pavillard F Djanikian F Dargazanli C Molinari N. Long-term outcome in patients with aneurysmal subarachnoid hemorrhage requiring mechanical ventilation. Dryad Dataset (2021) 16:e0247942. doi: 10.5061/dryad.47d7wm3b4, PMID: 33711023 PMC7954305

[9] Frontera JA Fernandez A Schmidt JM Claassen J Wartenberg KE Badjatia N. Defining vasospasm after subarachnoid hemorrhage: what is the most clinically relevant definition? Stroke (2009) 40:1963–8. doi: 10.1161/STROKEAHA.108.544700 19359629

[10] Vergouwen MDI Vermeulen M van Gijn J Rinkel GJE Wijdicks EF Muizelaar JP. Definition of delayed cerebral ischemia after aneurysmal subarachnoid hemorrhage as an outcome event in clinical trials and observational studies: proposal of a multidisciplinary research group. Stroke (2010) 41:2391–5. doi: 10.1161/STROKEAHA.110.589275, PMID: 20798370

[11] Zou KH Yu C-R Liu K Carlsson MO Cabrera J. Optimal thresholds by maximizing or minimizing various metrics via ROC-type analysis. Acad Radiol (2013) 20:807–15. doi: 10.1016/j.acra.2013.02.004 23582776

[12] Sauerbrei W Royston P Binder H. Selection of important variables and determination of functional form for continuous predictors in multivariable model building. Stat Med (2007) 26:5512–28. doi: 10.1002/sim.3148, PMID: 18058845

[13] Steyerberg EW Vergouwe Y. Towards better clinical prediction models: seven steps for development and an ABCD for validation. Eur Heart J (2014) 35:1925–31. doi: 10.1093/eurheartj/ehu207, PMID: 24898551 PMC4155437

[14] Collins GS Reitsma JB Altman DG Moons KGM. Transparent reporting of a multivariable prediction model for individual prognosis or diagnosis (TRIPOD): the TRIPOD statement. BMJ (2015) 350:g7594. doi: 10.1136/bmj.g7594 25569120

[15] Steyerberg EW Vickers AJ Cook NR Gerds T Gonen M Obuchowski N. Assessing the performance of prediction models: a framework for traditional and novel measures. Epidemiology (2010) 21:128–38. doi: 10.1097/EDE.0b013e3181c30fb2, PMID: 20010215 PMC3575184

[16] Vickers AJ Elkin EB. Decision curve analysis: a novel method for evaluating prediction models. Med Decis Mak an Int J Soc Med Decis Mak (2006) 26:565–74. doi: 10.1177/0272989X06295361, PMID: 17099194 PMC2577036

[17] Van Calster B Wynants L Verbeek JFM Verbakel JY Christodoulou E Vickers AJ. Reporting and interpreting decision curve analysis: a guide for investigators. Eur Urol (2018) 74:796–804. doi: 10.1016/j.eururo.2018.08.038, PMID: 30241973 PMC6261531

[18] Huang H-Y Yuan B Chen S-J Han Y-L Zhang X Yu Q. A novel nomogram model for clinical outcomes of severe subarachnoid hemorrhage patients. Front Neurosci (2022) 16:1041548. doi: 10.3389/fnins.2022.1041548, PMID: 36507324 PMC9729550

[19] Deng J He Z. Characterizing risk of in-hospital mortality following subarachnoid hemorrhage using machine learning: a retrospective study. Front Surg (2022) 9:891984. doi: 10.3389/fsurg.2022.891984, PMID: 36034376 PMC9407038

[20] Zhuang D Ren Z Sheng J Zheng Z Peng H Ou X. A dynamic nomogram for predicting unfavorable prognosis after aneurysmal subarachnoid hemorrhage. Ann Clin Transl Neurol (2023) 10:1058–71. doi: 10.1002/acn3.51789, PMID: 37198730 PMC10351672

[21] Li S Zhang J Li N Wang D Zhao X. Predictive nomogram models for unfavorable prognosis after aneurysmal subarachnoid hemorrhage: analysis from a prospective, observational cohort in China. CNS Neurosci Ther (2023) 29:3567–78. doi: 10.1111/cns.14288, PMID: 37287438 PMC10580355

[22] Zhou Z Liu Z Yang H Zhang C Zhang C Chen J. A nomogram for predicting the risk of poor prognosis in patients with poor-grade aneurysmal subarachnoid hemorrhage following microsurgical clipping. Front Neurol (2023) 14:1146106. doi: 10.3389/fneur.2023.1146106, PMID: 37034089 PMC10073426

[23] Nieuwkamp DJ Setz LE Algra A Linn FHH de Rooij NK Rinkel GJE. Changes in case fatality of aneurysmal subarachnoid haemorrhage over time, according to age, sex, and region: a meta-analysis. Lancet Neurol (2009) 8:635–42. doi: 10.1016/S1474-4422(09)70126-7, PMID: 19501022

[24] Huang H Lai LT. Incidence and case-fatality of aneurysmal subarachnoid hemorrhage in Australia, 2008-2018. World Neurosurg (2020) 144:e438–46. doi: 10.1016/j.wneu.2020.08.186, PMID: 32889187

[25] Schuss P Hadjiathanasiou A Borger V Wispel C Vatter H Güresir E. Poor-grade aneurysmal subarachnoid hemorrhage: factors influencing functional outcome – a single-center series. World Neurosurg (2016) 85:125–9. doi: 10.1016/j.wneu.2015.08.046, PMID: 26341439

[26] Maragkos GA Enriquez-Marulanda A Salem MM Ascanio LC Chida K Gupta R. Proposal of a grading system for predicting discharge mortality and functional outcome in patients with aneurysmal subarachnoid hemorrhage. World Neurosurg (2019) 121:e500–10. doi: 10.1016/j.wneu.2018.09.148 30268551

[27] Udy AA Vladic C Saxby ER Cohen J Delaney A Flower O. Subarachnoid hemorrhage patients admitted to intensive Care in Australia and new Zealand: a multicenter cohort analysis of in-hospital mortality over 15 years. Crit Care Med (2017) 45:e138–45. doi: 10.1097/CCM.0000000000002059 27749342

[28] Claassen J Vu A Kreiter KT Kowalski RG Du EY Ostapkovich N. Effect of acute physiologic derangements on outcome after subarachnoid hemorrhage. Crit Care Med (2004) 32:832–8. doi: 10.1097/01.ccm.0000114830.48833.8a, PMID: 15090970

[29] Kilbourn KJ Ching G Silverman DI McCullough L Brown RJ. Clinical outcomes after neurogenic stress induced cardiomyopathy in aneurysmal sub-arachnoid hemorrhage: a prospective cohort study. Clin Neurol Neurosurg (2015) 128:4–9. doi: 10.1016/j.clineuro.2014.10.017 25462088

[30] Zhao B Fan Y Xiong Y Yin R Zheng K Li Z. Aneurysm rebleeding after poor-grade aneurysmal subarachnoid hemorrhage: predictors and impact on clinical outcomes. J Neurol Sci (2016) 371:62–6. doi: 10.1016/j.jns.2016.10.020, PMID: 27871451

[31] Stienen MN Germans M Burkhardt J-K Neidert MC Fung C Bervini D. Predictors of in-hospital death after aneurysmal subarachnoid hemorrhage: analysis of a Nationwide database (Swiss SOS [Swiss study on aneurysmal subarachnoid hemorrhage]). Stroke (2018) 49:333–40. doi: 10.1161/STROKEAHA.117.019328 29335333

[32] Larsen CC Astrup J. Rebleeding after aneurysmal subarachnoid hemorrhage: a literature review. World Neurosurg (2013) 79:307–12. doi: 10.1016/j.wneu.2012.06.023 22722033

[33] Starke RM Connolly ESJ. Rebleeding after aneurysmal subarachnoid hemorrhage. Neurocrit Care (2011) 15:241–6. doi: 10.1007/s12028-011-9581-0 21761274

[34] Schuss P Konczalla J Platz J Vatter H Seifert V Güresir E. Aneurysm-related subarachnoid hemorrhage and acute subdural hematoma: single-center series and systematic review. J Neurosurg (2013) 118:984–90. doi: 10.3171/2012.11.JNS121435, PMID: 23289820

[35] Chwajol M Starke RM Kim GH Mayer SA Connolly ES. Antifibrinolytic therapy to prevent early rebleeding after subarachnoid hemorrhage. Neurocrit Care (2008) 8:418–26. doi: 10.1007/s12028-008-9088-5, PMID: 18386187

[36] Baharoglu MI Germans MR Rinkel GJE Algra A Vermeulen M van Gijn J. Antifibrinolytic therapy for aneurysmal subarachnoid haemorrhage. Cochrane Database Syst Rev (2013) 2013::CD001245. doi: 10.1002/14651858.CD001245.pub2, 2013, PMID: 23990381 PMC8407182

[37] Serrone JC Maekawa H Tjahjadi M Hernesniemi J. Aneurysmal subarachnoid hemorrhage: pathobiology, current treatment and future directions. Expert Rev Neurother (2015) 15:367–80. doi: 10.1586/14737175.2015.1018892 25719927

[38] Cahill J Calvert JW Zhang JH. Mechanisms of early brain injury after subarachnoid hemorrhage. J Cereb blood flow Metab Off J Int Soc Cereb Blood Flow Metab (2006) 26:1341–53. doi: 10.1038/sj.jcbfm.9600283 16482081

[39] Li R Lin F Chen Y Lu J Han H Ma L. A 90-day prognostic model based on the early brain injury indicators after aneurysmal subarachnoid hemorrhage: the TAPS score. Transl Stroke Res (2023) 14:200–10. doi: 10.1007/s12975-022-01033-4, PMID: 35567655

[40] Macdonald RL Higashida RT Keller E Mayer SA Molyneux A Raabe A. Clazosentan, an endothelin receptor antagonist, in patients with aneurysmal subarachnoid haemorrhage undergoing surgical clipping: a randomised, double-blind, placebo-controlled phase 3 trial (CONSCIOUS-2). Lancet Neurol (2011) 10:618–25. doi: 10.1016/S1474-4422(11)70108-9, PMID: 21640651

[41] Rass V Helbok R. Early brain injury after poor-grade subarachnoid hemorrhage. Curr Neurol Neurosci Rep (2019) 19:78. doi: 10.1007/s11910-019-0990-3, PMID: 31468197 PMC6715808

